# Metabolite Fingerprinting Using ^1^H-NMR Spectroscopy and Chemometrics for Classification of Three Curcuma Species from Different Origins

**DOI:** 10.3390/molecules26247626

**Published:** 2021-12-16

**Authors:** Laela Hayu Nurani, Abdul Rohman, Anjar Windarsih, Any Guntarti, Florentinus Dika Octa Riswanto, Endang Lukitaningsih, Nurrulhidayah Ahmad Fadzillah, Mohamad Rafi

**Affiliations:** 1 Faculty of Pharmacy, Universitas Ahmad Dahlan, Yogyakarta 55281, Indonesia; laelafarmasi@yahoo.com (L.H.N.); any_guntarti@yahoo.co.id (A.G.); 2 Department of Pharmaceutical Chemistry, Faculty of Pharmacy, Universitas Gadjah Mada, Yogyakarta 55281, Indonesia; lukitaningsih_end@ugm.ac.id; 3 Center of Excellence Institute for Halal Industry and Systems (IHIS), Universitas Gadjah Mada, Yogyakarta 55281, Indonesia; 4 Research Division for Natural Product Technology (BPTBA), National Research and Innovation Agency (BRIN), Yogyakarta 55861, Indonesia; anjarwindarsih2@gmail.com; 5 Division of Pharmaceutical Analysis and Medicinal Chemistry, Faculty of Pharmacy, Universitas Sanata Dharma, Yogyakarta 55282, Indonesia; dikaocta@uad.ac.id; 6 International Institute for Halal Research and Training (INHART), International Islamic University Malaysia (IIUM), Kuala Lumpur 50728, Malaysia; nurrulhidayah@iium.edu.my; 7 Department of Chemistry, Faculty of Mathematics and Natural Sciences, IPB University, Bogor 16680, Indonesia; mra@apps.ipb.ac.id

**Keywords:** authentication, curcuma, ^1^H-NMR spectroscopy, chemometrics, metabolite fingerprinting

## Abstract

*Curcuma longa*, *Curcuma xanthorrhiza*, and *Curcuma manga* have been widely used for herbal or traditional medicine purposes. It was reported that turmeric plants provided several biological activities such as antioxidant, anti-inflammatory, hepatoprotector, cardioprotector, and anticancer activities. Authentication of the Curcuma species is important to ensure its authenticity and to avoid adulteration practices. Plants from different origins will have different metabolite compositions because metabolites are affected by soil nutrition, climate, temperature, and humidity. ^1^H-NMR spectroscopy, principal component analysis (PCA), and orthogonal projections to latent structures-discriminant analysis (OPLS-DA) were used for authentication of *C. longa*, *C. xanthorrhiza*, and *C. manga* from seven different origins in Indonesia. From the ^1^H-NMR analysis it was obtained that 14 metabolites were responsible for generating classification model such as curcumin, demethoxycurcumin, alanine, methionine, threonine, lysine, alpha-glucose, beta-glucose, sucrose, alpha-fructose, beta-fructose, fumaric acid, tyrosine, and formate. Both PCA and OPLS-DA model demonstrated goodness of fit (R^2^ value more than 0.8) and good predictivity (Q^2^ value more than 0.45). All OPLS-DA models were validated by assessing the permutation test results with high value of original R^2^ and Q^2^. It can be concluded that metabolite fingerprinting using ^1^H-NMR spectroscopy and chemometrics provide a powerful tool for authentication of herbal and medicinal plants.

## 1. Introduction

For hundreds of years, herbal medicines and their preparations have been widely used in folk medicines over the world. The preparation of herbal medicine preparations is typically presented either as single herbs or several herbs in a composite formulae, and it is reported that about 92% of herbal medicine formulas are a combination of less than 13 herbs [1,2]. Annually, the market growth of herbal products has increased in which the raw material for most herbal products come from South Asian and Southeast Asian countries, including Indonesia [3]. Products of natural origin, such as supplements, herbal products, or herbal preparations, are increasingly widespread and used to maintain health or for the treatment of minor diseases. However, natural is not necessarily synonymous with safe. The adulteration of herbal preparations, together with contamination, sophistication, and degradation is a problem of global interest. In recent years, public awareness on herbal authentication and species admixtures in the raw herbal has increased significantly, because the adverse consequences of adulterated herbal components on consumer safety has been recognized [4]. Adulteration in herbal medicine involves replacing botanical materials, diluting high quality herbal medicines with lower grade ones, and mislabeling herbal medicine. Therefore, it is essential to have monitoring and pharmacovigilance systems [5].

For species authentication of herbal medicine, the World Health Organization (WHO), the United States Food and Drug Administration (USFDA), and the European Medicines Agency (EMEA) have regulated that the identification of herbal medicines should be made to ensure their quality and to discriminate them from related species or adulterated samples [6]. Among herbal medicines components, Curcuma species including *C. longa* (turmeric), *C. xanthorrhiza* (Java Turmeric), and *C. manga* have been widely applied as medicinal plants for herbal or traditional medicine purposes [7]. These Curcumas have been reported to have some biological effects which are beneficial to human health including antioxidant, anticancer anti-inflammatory, hepatoprotector, cardioprotector, antibacterial activities, and wound healing [8,9,10].

Some analytical methods have been developed for analysis of Curcuma species. Most of the methods use chromatography-based methods such as high performance-thin layer chromatography (TLC) [11], high performance liquid chromatography (HPLC), ultra-high performance liquid chromatography (UHPLC) [12], gas chromatography-mass spectrometry (GC-MS) for analysis volatile compounds in Curcuma species [13], and liquid chromatography-mass spectrometry (LC-MS/MS) [14]. Chromatographic-based methods typically involved complex sample preparation technique and resulted huge number of responses which make difficulty in data analysis. Therefore, spectroscopic-based methods in combination with multivariate data analysis (MDA) or chemometrics were potential to be employed since this combination method was provided the way to analyze such an environmental big data [15]. Ultraviolet, visible, and vibrational spectroscopy (infrared and Raman) [16,17], and NMR spectroscopy [18] are widely reported for authentication of Curcuma species. NMR spectroscopy offers some advantages for authentication of medicinal plants such as fast time analysis, simple in sample preparation, high reproducibility, and high robust. Moreover, NMR spectroscopy can be used for simultaneous analysis either primary or secondary metabolites comprehensively in certain samples [19,20]. Combined with chemometrics of multivariate analysis such as principal component analysis (PCA), partial least square-discriminant analysis (PLS-DA), and orthogonal projections to latent structures-discriminant analysis (OPLS-DA) which can manage the huge data generated from NMR measurement, it becomes a powerful analytical tool for metabolite fingerprinting of medicinal plants [21,22]. Combination of ^1^H-NMR spectroscopy and chemometrics of PLS-DA and OPLS-DA has been used for authentication of Saffron adulteration [23]. ^1^H-NMR spectroscopy and chemometrics have also been used for authentication of *C. longa* adulterated with *C. manga* and *C. heyneana* [7,24]. Authentication of *C. xanthorrhiza* from *C. aeruginosa* has been successfully investigated using ^1^H-NMR and multivariate analysis [18]. However, study on authentication of Curcuma species from different origins using ^1^H-NMR spectroscopy is still limited. Therefore, the objective of this study was to use ^1^H-NMR spectroscopy in combination with chemometrics for authentication of *C. longa*, *C. xanthorrhiza*, and *C. manga* from different origins.

## 2. Results and Discussion

### 2.1. H-NMR Spectra Analysis

**Figure 1:**
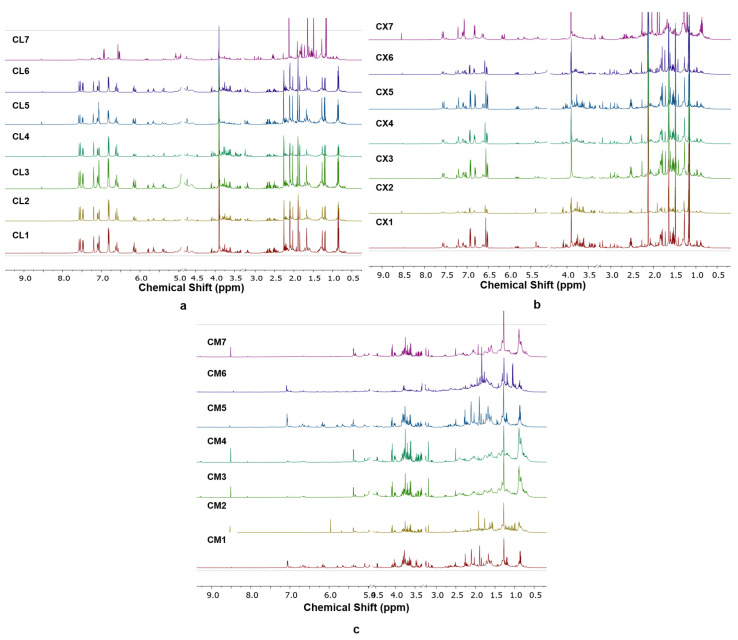
^1^H-NMR spectra of *C. longa* (**a**), *C. xanthorrhiza* (**b**), and *C. manga* (**c**) from different origins.

^1^H-NMR spectra can be used for authentication of medicinal plants because it offers fingerprinting which mean that each sample has specific ^1^H-NMR spectra pattern. Generally, metabolites of plants extracted using deuterated methanol and deuterium oxide measured using ^1^H-NMR spectroscopy are divided into three main regions, namely amino acid and organic acids (0.20–3.00 ppm), carbohydrate or sugar (3.01–5.00 ppm), and aromatic compounds (6.00–8.00 ppm) [25]. Different origins have different conditions such as soil condition, soil nutrition (macro and micronutrients), humidity, light, salinity, and temperature as well as internal developmental genetic circuits including regulated gene, and enzyme which can obviously affect the metabolite formation either primary or secondary metabolites [26]. The ^1^H-NMR spectra of *C. longa* (CL), *C. xanthorrhiza* (CX), and *C. manga* (CM) are shown in Figure 1. It can be observed that *C. longa*, *C. xanthorrhiza*, and *C. manga* have different spectra pattern indicating different metabolite contents. Specifically observed, *C. longa* and *C. xanthorrhiza* have higher signal intensities in the region of amino acid and organic acid (0.20–3.00 ppm) as well as in the aromatic region (6.00–8.00 ppm) than *C. manga*. On the other hand, the signal intensities in the region of glucose (3.01–5.00 ppm) are higher in *C. manga* compared to *C. longa* and *C. xanthorrhiza*. Fourteen metabolites in Curcuma species obtained from ^1^H-NMR measurement are shown in Table 1. Curcumin, demethoxycurcumin, alanine, methionine, threonine, lysine, alpha-glucose, beta-glucose, sucrose, alpha-fructose, beta-fructose, fumaric acid, tyrosine, and formate were stated as metabolites which play important roles in generating an OPLS-DA model. Investigation on each species obtained from different regions resulted in different signal patterns, especially in intensities, indicating the variations in metabolite contents in each species from different origins. It indicated that different origins affect the metabolite contents in each Curcuma rhizome. For example, *C. longa* from Blitar (CL7) has the lowest signal intensities in the aromatic region and *C. xanthorrhiza* from Gunungkidul (CX2) has the lowest signal intensities in the whole regions. However, the spectra patterns of each species are quite similar, therefore, for deeper classification of *C. longa*, *C. xanthorrhiza*, and *C. manga* from different regions powerful statistical tool such as chemometrics is required to obtain clear classification.

Curcuminoids have been reported as the active compound in Curcuma species. The content of curcuminoids is varied among Curcuma species and it is reported that curcuminoid content in *C. longa* and *C. xanthorrhiza* is higher among other Curcuma species. Curcuminoids consist of curcumin, demethoxycurcumin, and bisdemethoxycurcumin which curcumin possess the highest concentration. However, not all Curcuma species contain these three types of curcuminoids, for instance *C. xanthorrhiza* does not contain bisdemethoxycurcumin. Curcuminoids are aromatic molecules therefore most of the signals appeared in the chemical shift of aromatic regions. Curcumin signal could be observed in the chemical shift of 7.57 ppm (singlet), 7.28 ppm (singlet), 7.22 ppm (doublet), 6.77 ppm (doublet), and 3.90 ppm (singlet) whereas demethoxycurcumin could be found in the chemical shift of 6.92 ppm (doublet), 5.89 ppm (singlet), and 3.94 ppm (singlet) [28]. From the ^1^H-NMR spectra, higher signal intensities in the aromatic region of *C. longa* and *C. xanthorrhiza* supports that curcuminoids content in *C. longa* and *C. xanthorrhiza* is higher than in *C. manga*.

### 2.2. Chemometrics Analysis

**Figure 2:**
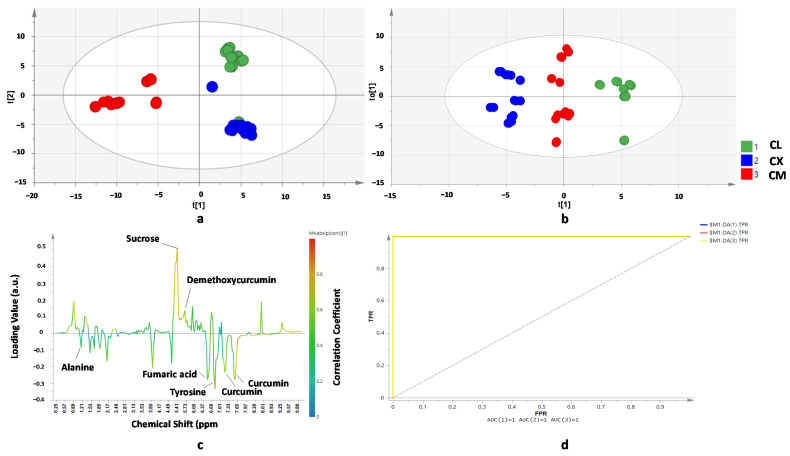
PLS-DA score plot (**a**), OPLS-DA score plot (**b**), OPLS-DA S-line correlation plot (**c**), and ROC curve (**d**) for differentiation and classification of *C. longa*, *C. xanthorrhiza*, and *C. manga* from different origins. Statistical parameters of the models: (**a**) Number of samples = 42; R^2^X = 0.603; R^2^Y = 0.783; Q^2^ = 0.755 for t[1] and t[2] components; (**b**,**c**) Number of samples = 42; R^2^X = 0.808; R^2^Y = 0.776; Q^2^ = 0.767 for t[1] and to[1] components.

Rhizomes of *C. longa*, *C. xanthorrhiza*, and *C. manga* are often used in a powder form as well as in an extract form for their herbal and traditional medicine applications. Both powder and extract are susceptible to adulteration because of their similar appearance especially in the adulterated form it is challenging to state whether the unknown sample is authentic or adulterated [29]. Chemometrics of PCA could not differentiate *C. longa*, *C. xanthorrhiza*, and *C. manga* clearly (data not shown). It might be caused by the large variations of the variables; therefore, the principal components (PC) were not able to represent the original variables. Observation using supervised pattern recognition, namely PLS-DA using 7 PC, could classify *C. longa*, *C. xanthorriza*, and *C. manga* resulting in three different classifications. However, several misclassifications occurred between *C. longa* and *C. xanthorrhiza* (Figure 2a). In the PLS-DA score plot, several *C. longa* samples appear in the region of *C. xanthorrhiza* and several *C. xanthorrhiza* samples appear in the region of *C. longa*. It can be explained that some of the metabolite compositions of *C. longa* and *C. xanthorrhiza* were similar especially in curcuminoid contents in which curcumin and demethoxycurcumin were the major active compounds in *C. longa* and *C. xanthorrhiza*. In addition, it is often reported that adulteration or substitution of *C. longa* with *C. xanthorrhiza* is often difficult to detect because the appearance of *C. longa* and *C. xanthorrhiza* in powder and extract form are quite similar [17]. Therefore, another supervised pattern recognition chemometrics, namely OPLS-DA, was performed to obtain better classification of *C. longa*, *C. xanthorrhiza*, and *C. manga* extracts. The OPLS-DA model demonstrated good capability to differentiate three different species of *C. longa*, *C. heyneana*, and *C. manga* from different origins as shown in the OPLS-DA score plot (Figure 2b). The OPLS-DA model successfully classified *C. longa*, *C. xanthorrhiza*, and *C. manga* samples. The samples were successfully classified using first PC and first X-orthogonal components which accounted for 80.8% of the variance with R^2^X (cum) of 0.808, R^2^Y (cum) of 0.776, and Q^2^ (cum) of 0.767. A high value of R^2^X (cum) and R^2^Y (cum) (close to 1) indicated goodness of fit of the OPLS-DA model, whereas the value of Q^2^ greater than 0.45 indicated goodness of predictivity of the models [30]. The S-line correlation plot (Figure 2c) variables which have roles in the differentiation of *C. longa*, *C. xanthorrhiza*, and *C. manga*. It was found that alanine, curcumin, demethoxycurcumin, fumaric acid, sucrose, and tyrosine had p (corr) values of more than 0.5, indicating their important roles in separating samples. Moreover, using variable importance in projection (VIP) value, chemical shifts of 6.77, 3.89, 7.57, 6.81, 6.57, 7.21, 1.49, 6.49, 6.13, 0.85, 6.09, 5.29, 5.25, and 6.92 ppm were found to have important roles for the classification between three Curcuma species in OPLS-DA models. Variables with VIP values greater than 1 are considered to have important roles for differentiation. Some of the variables correspond to the metabolites of curcumin, tyrosine, fumaric acid, alanine, and demethoxycurcumin. The receiver operating characteristic curve (Figure 2d) for differentiating and classifying Curcuma longa, Curcume xanthorrhiza, and Curcuma manga from different origins was also depicted. The ROC analysis represents the probability of the model by plotting the value of true positivity rate (TPR) against the value of false positivity rate (FPR) [31].

**Figure 3:**
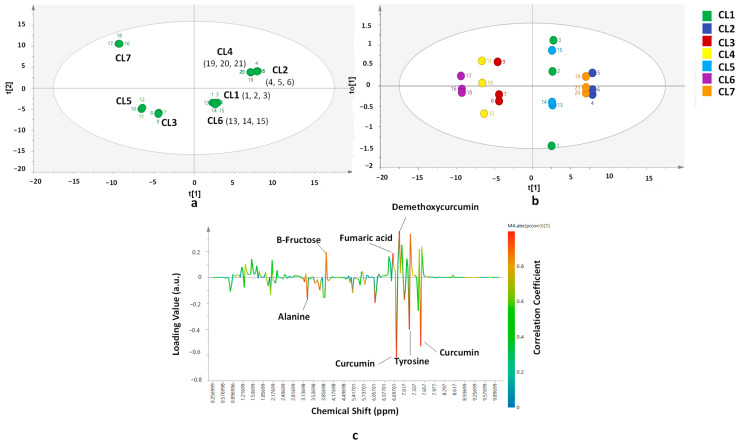
PCA score plot (**a**) OPLS-DA score plot (**b**) and OPLS-DA S-line correlation plot (**c**) for differentiation and classification *of C. longa* from different origins. Statistical parameter of the models: (**a**) Number of samples = 21; Number of PC = 8; R^2^X = 0.770; Q^2^ = 0.650; (**b**,**c**) Number of samples = 21; R^2^X = 0.748; R^2^Y = 0.754; Q^2^ = 0.639 for t[1] and to[1] components.

PCA using number of PC 8 could differentiate *C. longa* from seven different origins as shown in the PCA score plot (Figure 3a). The PCA model provided high confidence for its fitting and predictivity capacity, shown by its R^2^ value (0.770) and Q^2^ value (0.650) for PC1 and PC2, respectively, accounting for 77.0% of the variance. The score plots which appear close to each other indicates high similarity between samples, especially their metabolite compositions. The PCA score plot result shows that CL2 and CL4 possessed high similarity and CL1 has high similarity with CL6. Meanwhile, CL3 appeared closely to CL5. CL7 appeared far from all CL samples from other regions meaning that the metabolites composition of CL7 differs from other *C. longa* used in this research. Classification of *C. longa* samples from seven different origins using OPLS-DA demonstrated different pattern with PCA result (Figure 3b). OPLS-DA was created using first PC and first orthogonal-X component resulting R^2^X (cum) of 0.748, R^2^Y (cum) of 0.754 and Q^2^ (cum) of 0.639. The first PC and first X-orthogonal component explained 74.8% of the total variance. There were three main groups obtained from OPLS-DA classification. The first group was CL2 and CL7 which appeared close to each other. The second group consisted of CL1 and CL5, and the last group of CL3, CL4, and CL6. The important variables for differentiation and classification of *C. longa* between groups observed using S-line correlation plot (Figure 3c) were alanine, β-fructose, curcumin, demethoxycurcumin, fumaric acid, and tyrosine. Meanwhile, investigation using VIP value found that variables of 6.92, 6.54, 6.57, 1.50, 1.66, 1.54, 1.18, 7.46, 6.62, 0.86, 7.10, 7.50, 7.57, and 7.14 ppm had important roles for *C. longa* differentiation and classification from seven different origins classified using OPLS-DA model. Some of the variables correspond to the molecule signals of curcumin, demethoxycurcumin, fumaric acid, lysine, and tyrosine. It demonstrated that different origins affect the composition of some metabolites. It is in accordance with research by Jung et al. [27] on the metabolite compositions of *C. longa* from several regions in China. The condition of geographical origin and environmental conditions such as temperature, humidity, and rainfall rate affect the metabolite composition of plants.

**Figure 4:**
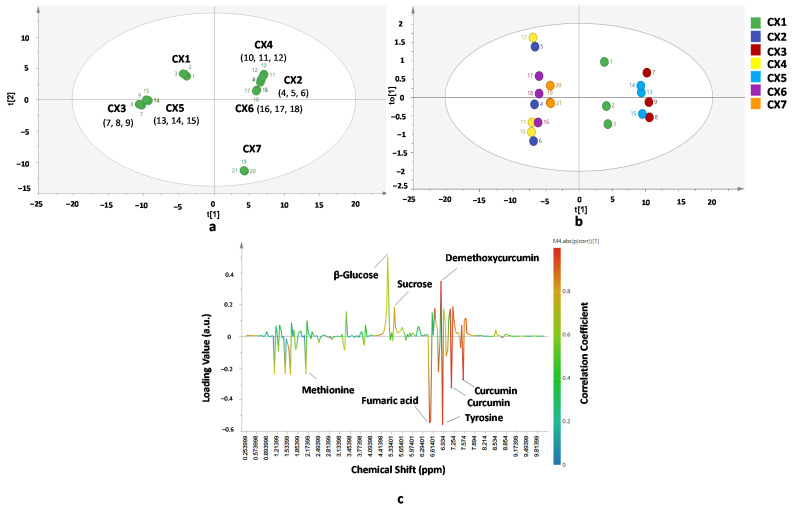
PCA score plot (**a**), OPLS-DA score plot (**b**), and OPLS-DA S-line correlation plot (**c**) for differentiation and classification *of C. xanthorrhiza* from different origins. Statistical parameter of the models: (**a**) Number of samples = 21; Number of PC = 6; R^2^X = 0.743; Q^2^ = 0.678; (**b**,**c**) Number of samples = 21; R^2^X = 0.743; R^2^Y = 0.833; Q^2^ = 0.626 for t[1] and to[1] components.

Chemometrics of PCA and OPLS-DA was also successfully used for differentiation and classification of *C. xanthorrhiza* from seven different origins. The result of differentiation of samples using PCA was slightly different with the result from OPLS-DA. PCA performed using first and second principal components demonstrated goodness of fit (R^2^cum = 0.743) and predictivity (Q^2^cum = 0.678) with total variance of 74.3%.Meanwhile, OPLS-DA was created using first PC and first orthogonal-X components. A high value of R^2^X (cum) (0.743) and R^2^Y (cum) (0.833) indicated good model fitness while a high value of Q^2^ (cum) (0.626) indicated predictivity of the OPLS-DA model. The first PC and first X-orthogonal-X component demonstrated 74.3% of the total variance. There were four main groups which appeared in the PCA score plot (Figure 4a), whereas in OPLS-DA (Figure 4b) there were three groups. *C. xanthorrhiza* of CX3 and CX5 appeared close to each other in both PCA and OPLS-DA score plot results. Meanwhile, *C. xanthorrhiza* of CX1 was found in a separate group with others observed both in PCA and OPLS-DA. Samples of CX2, CX4, CX6, and CX7 appeared in the same group observed using OPLS-DA; however, from the PCA result, a sample of CX7 appeared in a different group. From these results, it is suggested that different locations have significant effects on metabolites’ compositions in *C. xanthorrhiza*. The S-line correlation plot (Figure 4c) shows that methionine, β-glucose, sucrose, fumaric acid, curcumin, demethoxycurcumin, and tyrosine were the important variables for CX differentiation. Moreover, using a VIP value, it can be found that sthe variables important for classifying samples were 4.61, 6.57, 6.92, 4.59, 2.13, 6.92, 7.01, 7.21, 6.53, 1.69, 1.25, and 1.77 ppm. Some of the variables corresponded to the metabolites of curcumin, fumaric acid, demethoxycurcumin, and beta-glucose. It is presumed that these metabolites have higher scores and significantly affect the differentiation and classification of *C. xanthorrhiza* from different origins.

**Figure 5:**
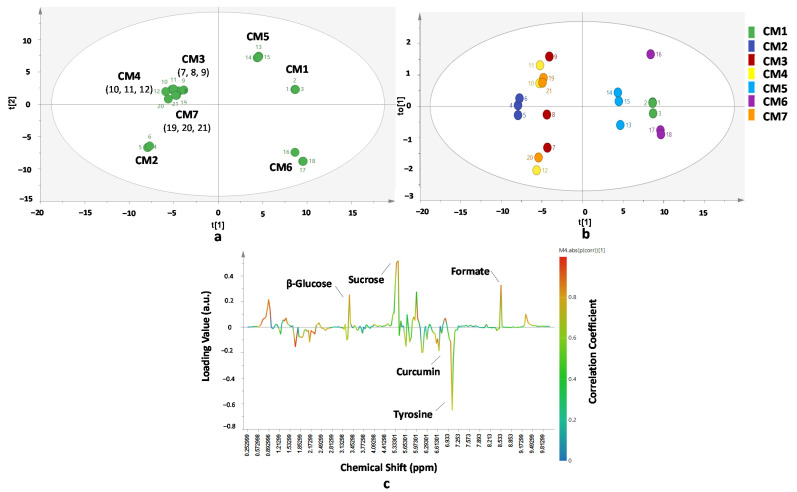
PCA score plot (**a**) OPLS-DA score plot (**b**) and OPLS-DA S-line correlation plot (**c**) for differentiation and classification *of C. manga* from different origins. Statistical parameter of the models: (**a**) Number of samples = 21; Number of PC = 7; R^2^X = 0.661; Q^2^ = 0.501; (**b**,**c**) Number of samples = 21; R^2^X = 0.661; R^2^Y = 0.667; Q^2^ = 0.707 for t[1] and to[1] components.

Chemometrics of PCA and OPLS-DA was also successfully applied for differentiation and classification of *C. manga* from seven different origins. Different classification results were observed between PCA and OPLS-DA. The PCA model was created using first and second principal components resulting R^2^X (cum) of 0.661 and Q^2^ (cum) of 0.501 indicating goodness of fit and good predictivity of the PCA model, respectively. The first and second PCs showed 66.1% of the total variance. From the PCA score plot (Figure 5a), *C. manga* were classified in five classes as follows: CM1 (first class), CM5 (second class), CM6 (third class), CM2 (fourth class), and the rest of the samples was in the last class (CM3, CM4, and CM7). Samples of CM3, CM4, and CM7 have similar chemical or metabolite compositions because they appeared in the same location in PCA score plot. It is presumed that the conditions in the region of Malang (CM3), Tulung Agung (CM4), and Blitar (CM7) are similar resulting in the similar metabolites of *C. manga* rhizomes. On the other hand, OPLS-DA was performed using first principal components and first orthogonal-X component which presented 66.1% of the total variance. The obtained R^2^X (cum) (0.661) and R^2^Y (cum) (0.667) indicated goodness of fit whereas the value of Q^2^ (cum) (0.707) demonstrated goodness of model predictivity. Three main groups were found in the OPLS-DA score plot (Figure 5b), namely CM1 and CM6 as the first group, CM5 in the second group, and CM2, CM3, CM4, and CM7 in the last group. Observation using an S-line correlation plot (Figure 5c) demonstrated that β-glucose, sucrose, curcumin, tyrosine, and format had important roles for CM differentiation. The VIP value showed that some variables were found to have significant contributions in the differentiation of *C. manga* samples from different origins, namely: 7.05, 5.29, 5.97, 5.33, 5.37, 8.53, 1.25, 1.81, 5.42, 5.65, 7.09, 6.17, 6.69, 0.85, 0.89, 3.33, and 5.41 ppm. Some of the variables are associated with curcumin, demethoxycurcumin, sucrose, and fumaric acid.

### 2.3. Validation of OPLS-DA Model. Using Permutation Test

**Figure 6:**
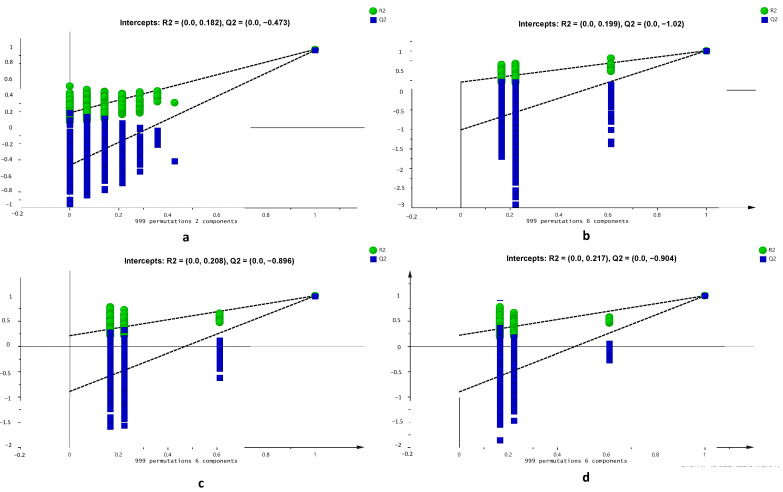
Permutation test using 999 permutations of OPLS-DA result from three Curcuma species (**a**), *C. longa* from different origins (**b**), *C. xanthorrhiza* from different origins (**c**), and *C. manga* from different origins (**d**).

Supervised pattern recognition of chemometrics such as PLS-DA and OPLS-DA requires a test to confirm the model’s validity because of its potential for overfitting. Validation is a confirmation step to ensure that the models have goodness of fit. A permutation test is one of validation testing which used a permutated model. Models of R^2^ and Q^2^ are permutated and compared to the original models of R^2^ and Q^2^. A good model is obtained when all the permutated models of R^2^ and Q^2^ values are lower than the R^2^ and Q^2^ original values. Moreover, the validation was also determined using intersection value of Q^2^. The intersection value should be zero or lower than zero to be categorized as valid models. The result of the permutation test from 999 permutations of OPLS-DA models were demonstrated in Figure 6. The permutated models of R^2^ and Q^2^ are on the left side while the original R^2^ and Q^2^ models are on the right side. The models were permutated for 100 permutations. Results showed that all permutation tests confirmed the validity of the OPLS-DA model demonstrated by the value of R^2^ and Q^2^ in all permutated models being below the value of original R^2^ and Q^2^ models. On the other hand, the intersection values of Q^2^ for all four OPLS-DA models were also zero and lower than zero, as follows: (0.0, −0.473) for a classification model between three Curcuma species; (0.0, −1.02) for classification model of *C. longa* from different origins; (0.0, −0.896), for classification model of *C. xanthorrhiza* from different origins; and (0.0, −0.904) for classification model of *C. manga* from different origins. It is suggested that OPLS-DA could be used as a powerful statistical tool for classification of different Curcuma species from different origins with high validity.

## 3. Materials and Methods

### 3.1. Sample Collection and Preparation

Rhizome of *C. longa* (CL), *C. xanthorrhiza* (CX), and *C. manga* (CM) were collected from seven different regions in Indonesia, namely: Boyolali (1), Gunungkidul (2), Ngawi (3), Malang (3), Tulung Agung (5), Karang Anyar (6), and Blitar (7). Determination of plant species used in this study has been carried out at the Pharmaceutical Biology Department, Faculty of Pharmacy, Universitas Gadjah Mada, Indonesia. Rhizomes were cleaned using running water then chopped into small pieces. Subsequently, the rhizomes were dried using an oven at 50 °C for 48 h. The dried rhizomes were then ground into powder.

### 3.2. Preparation of Curcuma Rhizome Methanolic Extract

The powdered rhizome of *C. longa*, *C. xanthorrhiza*, and *C. manga* were extracted using methanol pro analysis using sample to solvent ratio of 1:10. Extraction was performed using a maceration technique for 3 days. The supernatant was collected and evaporated using a vacuum rotary evaporator to obtain a concentrated methanolic extract.

### 3.3. ^1^H-NMR Analysis

Sample preparation was carried out according to Kim et al. [22] with modifications. An amount of 5 mg extract was weighed and placed into a 2 mL microtube. Subsequently the extract was added to 0.5 mL of deuterated methanol (CD_3_OD) and 0.5 mL of deuterium oxide (D_2_O) containing TMSP (trimethylsilyl propionic acid) 0.01%. The mixture was vortexed for 30 s and ultrasonicated for 20 min at room temperature. The sample was then centrifuged at 12,000 rpm for 10 min at room temperature. An amount of 800 µL of the supernatant was taken and transferred into an NMR tube. The sample was measured using a JEOL ECZ-R 500 MHz NMR spectrometer (JEOL, Tokyo, Japan). The NMR spectra acquisition was performed with the field strength of 11.74736 T, relaxation delay of 5 s, and X_offset of 5.0 ppm. Each spectrum was acquired for a 3.53 min acquisition time which consisted of 128 scans and a width of 12 ppm. Each sample was measured in three replicates.

### 3.4. Data Analysis

The ^1^H-NMR spectra were analyzed using MestreNova 12.0 Software (Mestrelab Research, S.L., Santiago de Compostela, Spain). Spectra were manually phase-corrected. Automatic baseline correction was performed using polynomial fit using degree of 3. The binning of the spectra was then performed for every 0.04 ppm from the chemical shift of 0.2–10 ppm excluding the region of residual water and methanol. Meanwhile, the chemometrics of multivariate analysis was performed using SIMCA 14.0 (Umetrics, Umeå, Sweden) software.

### 3.5. Chemometrics Analysis

Chemometrics of pattern recognition were used to analyze the data obtained from NMR measurements, namely principal component analysis (PCA), partial least square-discriminant analysis (PLS-DA), and orthogonal projections to latent structures-discriminant analysis (OPLS-DA). The data were processed using MestreNova 12.0 software (Mestrelab Research, S.L., Santiago de Compostela, Spain) for binning to extract the ^1^H-NMR data to obtain a dataset for chemometrics analysis. The data were normalized using total area. The variables used were the intensity values from the chemical shift of 0–10 ppm excluding the area of methanol and water residual. Prior to PCA, PLS-DA, and OPLS-DA analysis, Pareto scaling was performed to the dataset. The result was observed using a score plot, S-line correlation plot, variable importance in projection (VIP) value, and permutation test. Variables with a p (corr) value of more than 0.5 observed in an S-line correlation plot were important variables in OPLS-DA. Meanwhile, variables with a VIP value greater than 1 were considered to have important role in samples’ differentiation. In addition, in evaluation using a permutation test, the value of the original R2 and Q2 must have the highest value among permutated models.

## 4. Conclusions

Authentication of Curcuma species is important to ensure the quality, safety, and authenticity of the products. ^1^H-NMR spectroscopy method could be employed at the stage of sample fingerprinting for authentication purpose both for herbal and medicinal plants. Combined with chemometrics of PCA, PLS-DA, and OPLS-DA, ^1^H-NMR spectroscopy method is a powerful analytical tool for authentication of Curcuma species from different origins.

Exploratory data and classification models were successfully built. Several useful plots of output from the chemometrics models were also presented to visually assess the classification analysis. Predictive models for each species including *C. longa*, *C. xanthorrhiza*, and *C. manga* were evaluated according to the high values of the R^2^ and Q^2^. Other statistical and visual observations made considering the ROC curve and permutation test proved the probability and performance quality of the model. Hence, there is promise and potential to develop a combinational method with data fusion of ^1^H-NMR spectroscopy and chemometrics technique for the authentication of medicinal plants and herbal products.

## Figures and Tables

**Table 1:** Several metabolites of Curcuma species observed using ^1^H-NMR spectra obtained from this study. The assignment of the metabolites refers to the previous published literature by Jung et.al and Awin et al. [27,28].

No.	Chemical Shift (ppm)	Multiplicity	Metabolite
1.	7.57	Singlet	Curcumin
	7.28	Singlet	
	7.21	Doublet	
	6.77	Doublet	
	3.68	Singlet	
2.	6.92	Doublet	Demethoxycurcumin
	5.89	Singlet	
	3.94	Singlet	
3.	1.49	Doublet	Alanine
	3.72	Quartet	
4.	2.11	Singlet	Methionine
5.	1.33	Doublet	Threonine
	3.53	Doublet	
6.	3.81	Triplet	Lysine
	1.5	Multiplet	
7.	5.19	Doublet	Alpha-Glucose
	3.46	Doublet of Doublet	
	3.67	Triplet	
	3.35	Triplet	
8.	4.59	Doublet	Beta-Glucose
	3.19	Doublet of Doublet	
	3.44	Triplet	
	3.71	Doublet of Doublet	
9.	5.42	Doublet	Sucrose
	3.74	Triplet	
	3.43	Triplet	
	3.80	Multiplet	
	3.84	Multiplet	
10.	4.07	Doublet	Alpha-Fructose
	3.82	Doublet of Doublet	
	3.53	Doublet	
	3.55	Doublet	
	3.63	Quartet	
11.	3.95	Multiplet	Beta-Fructose
	3.52	Doublet	
	4.02	Doublet of Doublet	
12.	6.57	Singlet	Fumaric acid
13.	6.81	Doublet	Tyrosine
	7.14	Doublet	
14.	8.42	Singlet	Formate

## Data Availability

The data presented in this study are available in the article.

## Sample Availability

Samples of the extracts of *Curcuma longa*, *Curcuma xanthorrhiza* and *Curcuma manga* are available from the authors.
